# Exploring the sex-dependent effects of midnolin loss on hepatic transcription and chromatin accessibility

**DOI:** 10.1016/j.bbrep.2026.102745

**Published:** 2026-08-17

**Authors:** Lakshmi Sudeepthi Nissankararao, Gayeoun Kim, Soo-Mi Kweon, Vaisnavii Mohanraj, Damien Parrello, N. Sreekar, Sheng-Wei Chang, Walter Tsark, Keane Lai, Motoki Takaku

**Affiliations:** a Department of Biomedical Sciences, University of North Dakota School of Medicine and Health Sciences, Grand Forks, ND, 58202, USA; b Department of Pathology, University of Southern California, Los Angeles, CA, 90089, USA; c Department of Molecular Medicine, Beckman Research Institute, City of Hope, Duarte, CA, 91010, USA; d Center for Comparative Medicine, Beckman Research Institute, City of Hope, Duarte, CA, 91010, USA

**Keywords:** Chromatin accessibility, Gene regulation, Hepatocyte metabolism, Sex differences, RNA sequencing, ATAC sequencing, Ubiquitin-independent protein degradation

## Abstract

Midnolin (Midn) is a ubiquitin-like domain–containing nuclear protein that interacts with the proteasome and mediates ubiquitin-independent degradation of transcription factors. Although Midn has been implicated in pancreatic β-cell metabolism, immune regulation, and hepatocellular carcinoma (HCC) progression, its physiological role in normal liver remains poorly defined. To investigate Midn function in vivo, we generated a liver-specific Midn e**X**pressed/**T**rapped/**R**estored [XTR] conditional-by-inversion gene-trap mouse model. Transcriptomic and chromatin accessibility profiling were performed using RNA-seq and ATAC-seq in male and female livers. Midn loss resulted in sex-dependent transcriptional alterations. In male liver, differential expression analysis revealed prominent changes in lipid-associated pathways, including fatty acid biosynthesis, steroid metabolism, and retinoic acid and bile acid metabolism. In females, fewer but biologically coherent transcriptional changes were observed, with enrichment of metabolic and membrane-associated gene categories. ATAC-seq in female liver identified modest changes in chromatin accessibility and enrichment of transcription-factor motifs associated with nuclear receptor and hepatic metabolic regulatory programs, whereas exploratory analysis in male liver using a less stringent statistical threshold suggested potential accessibility changes involving similar programs related to hepatocyte identity and metabolism. Together, these findings suggest that Midn contributes to hepatic transcriptional regulation and may influence chromatin accessibility in a sex-dependent manner. Our study provides insight into Midn function in liver homeostasis and establishes a foundation for investigating how Midn-dependent proteasomal control may contribute to metabolic liver disease and HCC.

## Highlights

• Midnolin loss alters hepatic gene expression in a sex-dependent manner.
• ATAC-seq reveals changes in liver chromatin accessibility upon Midn loss.
• Metabolic and transporter pathways are dysregulated in Midn-deficient liver.
• Midnolin links transcription factor regulation to hepatic gene programs.

## List of abbreviations:

**ATAC-seq**–: Assay for Transposase-Accessible Chromatin using sequencing
**BP**–: Biological Process (Gene Ontology)
**CC**–: Cellular Component (Gene Ontology)
**ChEA3**–: ChIP-X Enrichment Analysis 3
**COIN**–: Conditional by Inversion
**DEG**–: Differentially Expressed Gene
**FDR**–: False Discovery Rate
**GO**–: Gene Ontology
**GSEA**–: Gene Set Enrichment Analysis
**HOMER**–: Hypergeometric Optimization of Motif EnRichment
**KEGG**–: Kyoto Encyclopedia of Genes and Genomes
**MF**–: Molecular Function (Gene Ontology)
**Midn**–: Midnolin
**NES**–: Normalized Enrichment Score
**PCA**–: Principal Component Analysis
**RNA-seq**–: RNA sequencing
**TF**–: Transcription Factor
**WT**–: Wild Type
**XTR**–: eXpressed-Trapped-Restored

## 1 Introduction

Protein degradation is a fundamental process required to maintain cellular homeostasis, regulate signaling pathways, and ensure proper turnover of structural and regulatory proteins [[1], [2], [3]]. In eukaryotes, the ubiquitin–proteasome system (UPS) constitutes the major pathway for targeted protein degradation, where ubiquitin conjugation serves as the canonical signal for proteasome recruitment and substrate destruction [[4], [5], [6], [7], [8]]. More recent work, however, has revealed the existence of ubiquitin-independent degradation mechanisms that expand our understanding of proteasome specificity [[9], [10], [11], [12], [13], [14]]. One such mechanism is the midnolin–proteasome pathway, which mediates the degradation of nuclear proteins—including unstable transcription factors encoded by immediate-early genes (EGR1, FosB, c-FOS), without requiring ubiquitin conjugation [15]. Midnolin (Midn) contains a ubiquitin-like (Ubl) domain that binds directly to the proteasome and a “Catch” domain required for substrate recognition; mutations in this domain disrupt its degradation activity [15].

Midn was initially described as a nucleolar protein enriched in the developing mesencephalon [16], but subsequent studies established broader roles in cellular metabolism and disease, including cancer [[17], [18], [19]]. Midn modulates glucokinase function through its Ubl domain in pancreatic β-cells [20] and regulates parkin expression in neuronal cells [21]. In the liver, Midn expression is elevated in hepatocellular carcinoma (HCC), promotes tumorigenicity, and influences retinoic acid and lipid metabolism [22,23]. Genetic studies further indicate that Midn plays an essential role in immune development, with Midn deficiency altering lymphocyte populations and proteasome activity [24]. Despite these emerging functions, the role of Midn in regulating nuclear chromatin architecture and transcription in the liver remains unclear. Previous mouse models with constitutive Midn deficiency showed strong expression in the midbrain of e12.5 fetuses and perinatal lethality in homozygous mutants, which precluded comprehensive studies in adult mice [16,22,24]. To address this gap, we generated a liver-specific Midn Cre recombinase inducible gene-trap mouse model and performed genome-wide analyses of gene expression and chromatin accessibility in both sexes. Our findings uncover sex-specific transcriptional consequences of Midn loss and reveal that Midn regulates chromatin accessibility and gene expression programs associated with lipid metabolism, providing new insight into its physiological role in liver homeostasis.

## 2 Methods

### 2.1 Midn XTR (eXpressed-Trapped-Restored) conditional gene-trap mouse

All animal experiments were performed in compliance with relevant laws and institutional guidelines and approved by the Institutional Animal Care and Use Committee (IACUC Protocol #18153) at City of Hope. A conditional Midn depletion mouse model was generated using the COIN (Conditional by Inversion) gene-trap strategy [25,26], implemented with an XTR (eXpressed-Trapped-Restored) cassette [27] inserted into intron 2–3 of the Midn gene. Several CRISPR guide RNAs targeting intron 2–3 of Midn were designed using the MIT CRISPR design tool [28]. The activity of each CRISPR single guide RNA (sgRNA; Synthego, Redwood City California USA) was tested in mouse embryos [29], and Midn In23 CR2 sgRNA (5′-GCTCGTCTTTGTGGGCGCCG-3′) was selected. Midn-XTR gene-trap plasmid was constructed as follows. A GBlock dsDNA fragment (IDT) with Midn intron 2–3 flanking the CRISPR gRNA target site, with upstream (78 bp) and downstream (79 bp) homology arms, separated by BstBI and AvrII restriction sites, was subcloned into pCR-BluntII-TOPO (Invitrogen) using terminal HindIII and *Xba*I restriction sites to create pCR-mMidn-GBlock. A BstBI/AvrII fragment containing the XTR gene-trap cassette was isolated from plasmid pNeoXTR f0 (Addgene #69157, a gift from David Feldser [27]) and ligated into BstBI/AvrII [**sites in bold type**] -digested pCR-mMidn-GBlock between the mMidn homology arms [underlined] AGCTAAGCTTTAATTAACCCTCACTAAAGGATATCCAGCGGGCCTGCGCTGGCGAGGGAGGAGGGGGAGCCTGGGTCGGCAGAGCCCCCGAAGGCCTCTCAAAGAGGGCCGGAG**TTCGAA**GTATTCC**CTAGG**CCCACAAAGACGAGCCCGGGCAAGAATTGGAGAGTTGTAGGCAGGAGCTGGCCTGAAGAGAGGCGCGGGGGAGGGTGGCTCTAGAGCTATC to create mMidn_XTR_DonorTemplate plasmid. Biotin-labeled PCR products from multiple reactions with Midnolin XTR donor template plasmid using primers biotin-M13F x (5′-Biotin-GTAAAACGACGGCCAGT-3′) and M13R (5′-CAGGAAACAGCTATGACC-3′) were combined and purified. Poly-adenylated Cas9-monomeric Streptavidin (Cas9-mSA) mRNA was in vitro transcribed from the SP6 promoter of pCS2+Cas9-mSA (Addgene #103882, a gift from Janet Rossant [30]), linearized with *Xba*I (Ambion SP6 mMESSAGE mMACHINE + pA kit). Homologous recombination was performed in 2-cell-stage C57BL/6J mouse embryos injected with Cas9–monomeric streptavidin mRNA, CRISPR single-guide RNA, and biotinylated double-stranded DNA repair template [30,31]**.** Female C57BL/6J mice were superovulated as described previously [32]. Embryos were collected at the 2-cell stage and each blastomere of C57BL/6J was microinjected with 75 ng/μl Cas9-mSA mRNA and 20 ng/μl mMidn In23 XTR gene-trap BioPCR template in 1 mM Tris/0.1 mM EDTA/50 mM NaCl using a Micro-ePore pinpoint cell penetrator (World Precision Instruments, Sarasota FL USA). The microinjected embryos were transferred to the oviducts of pseudopregnant female mice and the resulting founder mice were genotyped to identify those carrying the Midn-XTR gene-trap allele. In the eXpressed configuration, the cassette resides in the noncoding orientation and includes a GFP reporter and splice acceptor sequence positioned antisense to the endogenous transcript, allowing normal transcription and splicing of Midn exons 1–8 under baseline conditions. Exposure to Cre recombinase induces an irreversible inversion of the cassette into the Trapped configuration, placing the splice acceptor and GFP reporter in the sense orientation. This redirects endogenous Midn transcription to terminate prematurely after exon 2 and produce a GFP fusion transcript, thereby disrupting Midn expression while marking recombined cells by GFP fluorescence. Because transcription is interrupted upstream of the cassette, the resulting Midn transcript retains exons 1–2, whereas sequences downstream of exon 2 are excluded; any aberrant exon 2–8 fusion product would be expected to undergo nonsense-mediated decay. The XTR design allows cell type-specific or tissue-specific expression patterns of eXpressed (Midn-X) and Trapped (Midn-T) alleles depending on the specificity and activity of the Cre driver. Expression of Flpe recombinase will excise the gene-trap cassette fully and restore expression of the target gene. For the present study, mice harboring the Midn-XTR allele were crossed with a liver-specific Albumin-Cre line, Cg-Speer6-ps1Tg (Alb-cre)21Mgn/J, enabling Midn inactivation selectively in hepatocytes. Liver tissues were collected from mice aged 11–14 weeks, snap-frozen in liquid nitrogen, and stored at −80 °C.

### 2.2 RNA purification and RNA-seq

Total RNA was isolated from mouse liver tissue (N = 3) using the RNeasy Mini Kit (Qiagen) according to the manufacturer's protocol for animal tissues. Briefly, tissues were homogenized in Buffer RLT supplemented with β-mercaptoethanol and clarified by centrifugation. Following the addition of 70% ethanol, RNA was bound to the silica membrane, washed using the provided wash buffers, and eluted in RNase-free water. RNA concentration and purity were assessed using a NanoDrop spectrophotometer, and RNA integrity was evaluated by Agilent Bioanalyzer analysis.

For library preparation, ribosomal RNA (rRNA) was depleted from 25 to 1000 ng of total RNA using the KAPA RNA HyperPrep Kit with RiboErase (Roche). rRNA depletion was achieved by hybridization of rRNA to complementary DNA oligonucleotides, followed by RNase H and DNase treatment to remove rRNA–DNA hybrids. The remaining RNA was fragmented using heat and magnesium, and first-strand cDNA synthesis was performed using random priming. Second-strand synthesis incorporated dUTPs into the cDNA. Sequencing adapters were ligated, and libraries were PCR-amplified. Library size distribution was assessed using an Agilent Bioanalyzer.

RNA purification, library preparation, and sequencing were performed at the Yale Center for Genome Analysis (YCGA). All libraries were sequenced on a NovaSeq 6000 (Illumina) platform to generate paired-end reads (150 bp). Sequencing reads were aligned to the *Mus musculus* GRCm39 reference genome (mm39) using STAR [33] with default parameters for stranded paired-end RNA-seq. Gene annotation was based on the Ensembl release 110 (Mus_musculus.GRCm39.110.chr.gtf). Following alignment, uniquely mapped reads were assigned to genes using HTSeq [34] in reverse-stranded mode. Gene-level count tables were generated for all samples and used for downstream differential expression analyses.

Differential expression analysis was performed using the DESeq2 [35] package in R. Count matrices were imported into DESeq2. Dispersion estimates and fold changes were calculated using DESeq2's default statistical framework. Pairwise contrasts were conducted to compare wild-type (WT) and Midn-T samples within male and female groups separately. Genes with an adjusted P value (FDR) < 0.1 were considered significantly differentially expressed (Supplementary Table 1). Functional enrichment analyses of differentially expressed genes were conducted using DAVID [36].

For qPCR, the following primers were used:

Midn: Forward 5′-GTTGTCCCAACGCCTCAAAG-3’ (exon 2), Reverse 5′-CAAGGCTTGCATAACGGACTG-3’ (exon 4).

EGFP: Forward 5′-GCTGACCCTGAAGTTCATCT-3′, Reverse 5′-AGAAGTCGTGCTGCTTCAT-3’.

### 2.3 ATAC-seq

ATAC-seq libraries were generated from frozen mouse liver tissues (N = 2) at the YCGA, following their standard ATAC-seq workflow. 20–30 mg of tissue was used as input for each library. Nuclei were isolated using the Singulator™ 200 system (S2 Genomics) according to the manufacturer's protocol and counted using Trypan Blue staining. For each sample, 50,000 nuclei were used for library preparation with the ATAC-Seq Kit (Active Motif) following the manufacturer's instructions. Intact nuclei were subjected to transposition with Tn5 transposase, and adapter-ligated DNA fragments were PCR-amplified using i7 and i5 indexed primers. The size distribution of the final libraries was assessed using an Agilent TapeStation system, and library quantification was performed by quantitative PCR using the KAPA Library Quantification Kit (Roche). Libraries were sequenced on an Illumina NovaSeq 6000 platform to generate paired-end 150-bp reads. Sequencing reads were aligned to the *Mus musculus* GRCm39 (mm39) reference genome using STAR [33]. Duplicate reads were removed prior to peak calling. Peaks were identified using MACS2 [37,38], and peaks from all samples were merged to generate a unified peak set for downstream analyses.

Peak-level read counts were obtained using featureCounts [39] on the unified peak set, with each peak refined to a 500 bp window centered at the peak midpoint. Differential peak analysis between WT and Midn-T samples was performed using edgeR [40] with TMM normalization in the SARTools [41] package. For female mice, peaks with FDR < 0.1 were considered significantly differentially accessible (Supplementary Table 2). For male mice, raw P < 0.05 was used to identify exploratory differential peaks due to sample-to-sample variability (Supplementary Table 2). Motif enrichment analysis (de novo and known) was performed using HOMER [42], and peak-to-gene assignments were generated using ChIPpeakAnno [43].

### 2.4 Data integration between ATAC-seq and RNA-seq

Genes associated with differential ATAC-seq peaks were annotated to the nearest protein-coding gene. For each set of increased or decreased ATAC peaks, the corresponding gene lists were used as custom gene sets. RNA-seq differential expression results were ranked by the DESeq2 stat value, and gene-set enrichment analysis [44] was performed using the fgsea multilevel algorithm [45]. Normalized enrichment scores (NES), P values, and FDR values were obtained from fgsea, and leading-edge subsets were used to define core enriched genes. Enrichment plots were generated to visualize the trend of ATAC-associated genes across the ranked transcriptome.

## 3 Results

### 3.1 Loss of downstream exon usage and distinct transcriptional profiles in livers of mice with the Midn gene-trapped (Midn-T) allele

**Fig. 1:**
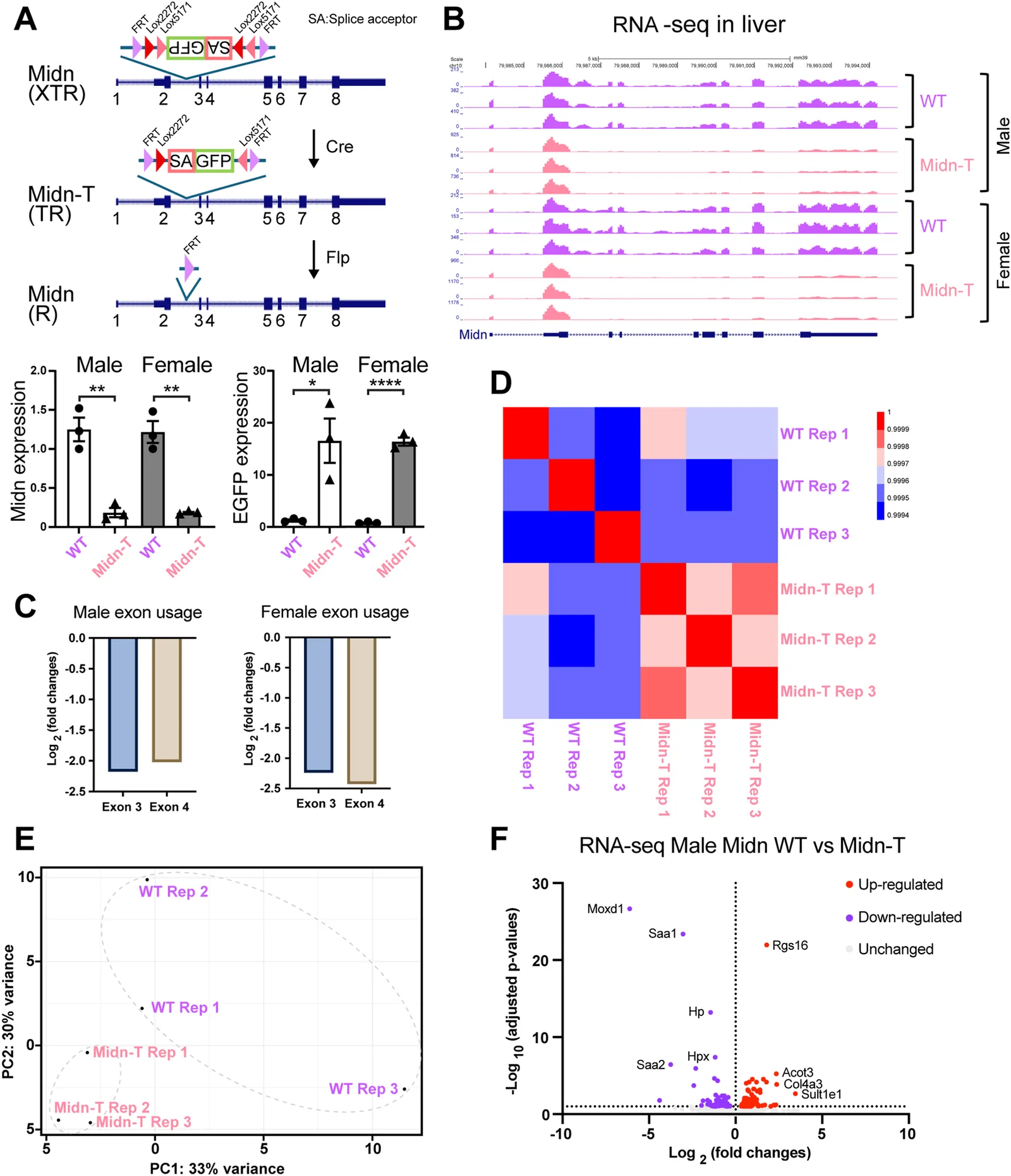
Midn conditional depletion strategy and transcriptomic changes in mouse liver **(A)** Schematic of the Midn genomic locus and qPCR results for Midn gene-trap confirmation. The COIN-based Midn conditional depletion allele is shown in the top panel. Insertion of an invertible GFP-containing gene-trap cassette into intron 2 disrupts Midn transcription upon Cre-mediated inversion. The XTR allele (eXpressed) is converted by Cre recombination into the TR (Trapped) state, resulting in gene disruption and GFP expression. This gene-trapped state can be restored to the R (Restored) state by Flp recombination. qPCR analysis confirmed a marked reduction of Midn expression in both male and female livers, accompanied by robust induction of GFP expression following gene-trap. Data are presented as mean ± SEM from three biological replicates per genotype for each sex. Statistical comparisons were performed using an unpaired two-tailed Student's t-test. *P < 0.05, **P < 0.01, and ****P < 0.0001. **(B)** RNA-seq genome browser tracks illustrating Midn expression across WT and Midn-T male and female liver samples. RNA-seq was performed using three biological replicates per genotype for each sex. WT animals show abundant reads across Midn, whereas Midn-T samples display truncation at exon 2 with no downstream transcription. **(C)** Midn exon usage analysis showing selective loss of exon 3 and 4 usage. **(D)** Heatmap of sample-to-sample correlation based on normalized RNA-seq counts in male liver. WT and Midn-T samples cluster separately. **(E)** Principal component analysis (PCA) of liver transcriptomes demonstrating clear separation between WT and Midn-T. **(F)** Volcano plot of differentially expressed genes (DEGs) in male Midn-T versus WT livers. Up-regulated genes in Midn-T are shown in red, down-regulated genes in blue, and unchanged genes in gray.

To interrogate the physiological role of Midn in the liver, we utilized a conditional gene-trap strategy in which an XTR COIN cassette containing a GFP reporter was inserted into intron 2 of the Midn locus. Cre-mediated inversion disrupts the normal transcriptional continuity between exon 2 and downstream exons, resulting in a truncated transcript while simultaneously activating GFP expression (Fig. 1A). This strategy is designed to eliminate Midn protein production by preventing splicing of exons 3-8, resulting in the absence of the conserved Ubiquitin-like domain. Quantitative PCR (qPCR) analysis confirmed a robust reduction of Midn mRNA levels in both male and female livers, accompanied by induction of GFP expression following gene-trap (Fig. 1A). To further validate the loss of Midn expression, we performed bulk RNA-seq on livers from male and female WT and Midn-T mice. Genome browser visualization revealed robust Midn transcription across all exons in WT samples, whereas Midn-T samples showed read coverage restricted to exons 1–2, with complete absence of downstream exons (Fig. 1B). Consistent with this observation, exon usage analysis demonstrated a selective loss of exon 3 and exon 4 usage in Midn-T samples relative to WT controls (Fig. 1C).

We next assessed global transcriptional patterns. Sample-to-sample correlation matrices showed tight clustering among biological replicates within each genotype and clear segregation between WT and Midn-T male samples (N = 3, Fig. 1D). Principal component analysis (PCA) further demonstrated strong genotype-dependent separation, with principal components 1 and 2 explaining 33% and 30% of the variance, respectively (Fig. 1E). These data indicate that Midn deficiency induces reproducible transcriptomic remodeling in both sexes. Differential expression analysis in male liver samples identified genes significantly altered by Midn loss, including both 108 up-regulated and 75 down-regulated genes at an FDR < 0.1 (Fig. 1F). Several metabolic and immune-related genes exhibited notable changes, suggesting that Midn plays a role in maintaining hepatic transcriptional homeostasis.

### 3.2 Midn deficiency alters hepatic metabolic and transporter-related pathways

**Fig. 2:**
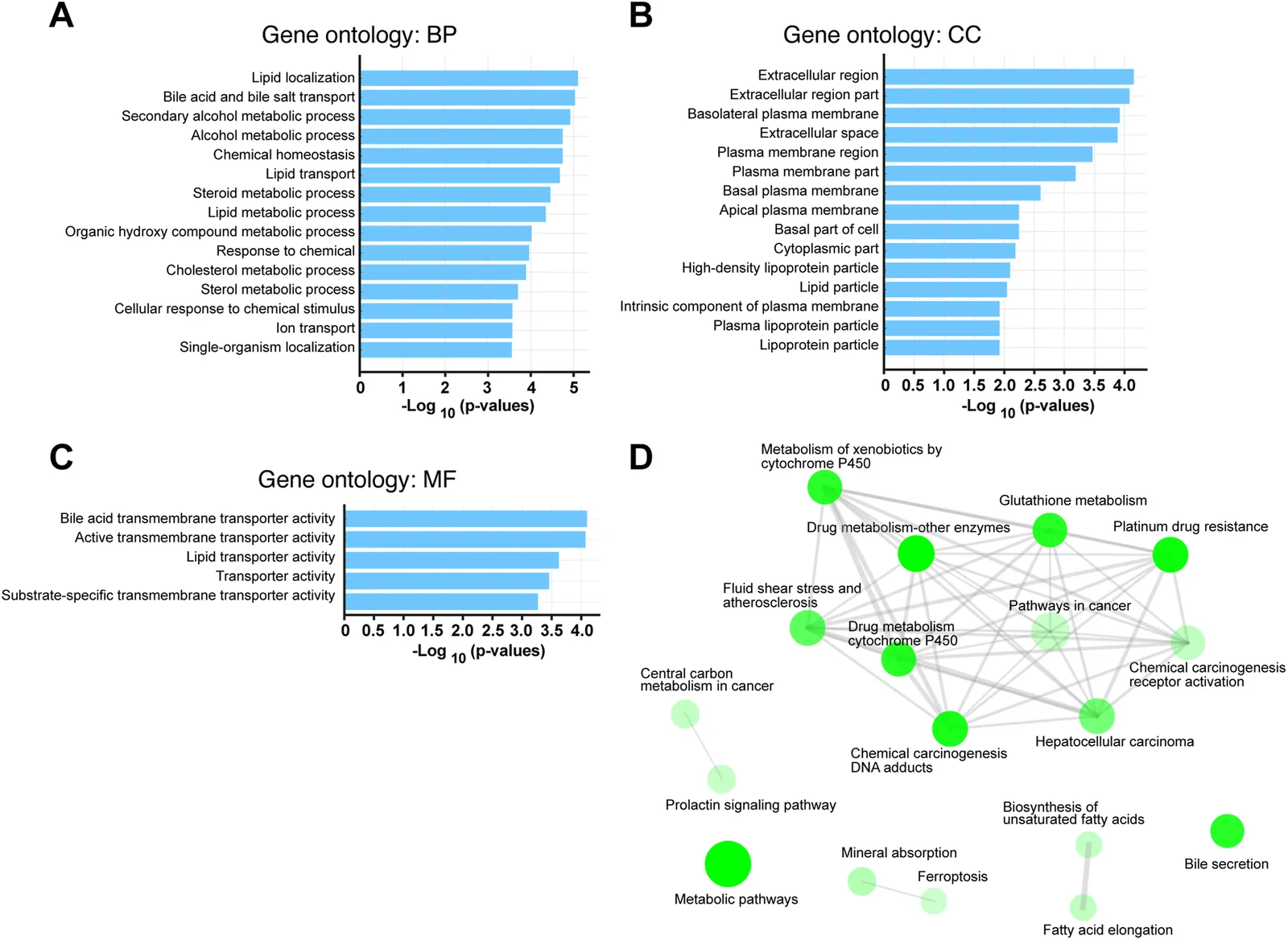
Gene Ontology (GO) enrichment of differentially expressed genes in Midn-T liver **(A)** Enriched Biological Process (BP) terms associated with DEGs in male livers. Lipid metabolic processes, steroid metabolism, and organic compound metabolism are among the top enriched pathways. **(B)** Enriched Cellular Component (CC) categories showing enrichment of extracellular region, plasma membrane regions, and lipoprotein particles. **(C)** Enriched Molecular Function (MF) categories, including bile acid and lipid transporter activity. **(D)** KEGG pathway similarity network constructed from enriched pathways based on gene set overlap (edge cutoff = 0.3). Node size reflects the number of differentially expressed genes per pathway, and node opacity indicates enrichment significance. Edges represent pathway similarity based on shared gene content.

To identify the biological processes affected by Midn loss, we performed functional enrichment analysis using the differentially expressed genes between male Midn wild-type and Midn-T livers. Gene Ontology Biological Process (GO BP) terms were significantly enriched for pathways involved in lipid metabolism, including lipid localization, bile acid and bile salt transport, alcohol and steroid metabolic processes, and chemical homeostasis (Fig. 2A). These findings indicate that Midn regulates a subset of metabolic genes essential for maintaining hepatic lipid and sterol balance. GO Cellular Component (GO CC) analysis revealed strong enrichment for extracellular regions, multiple plasma membrane subdomains, and lipoprotein particle-related compartments (Fig. 2B). These components align with the metabolic and secretory functions of hepatocytes and suggest that Midn loss impacts genes associated with membrane trafficking and extracellular metabolic signaling. GO Molecular Function (GO MF) enrichment further highlighted alterations in transmembrane transporter activity, including bile acid transmembrane transporter activity and lipid transporter activity (Fig. 2C). These results support the interpretation that Midn-T disrupts transport processes critical for hepatic bile acid handling and lipid flux.

To further contextualize these enrichment results at the pathway level, we constructed a KEGG pathway similarity network based on gene set overlap (edge cutoff = 0.3), enabling visualization of functionally related pathways as interconnected modules (Fig. 2D). This network analysis revealed a prominent, densely connected cluster enriched for xenobiotic and drug metabolism pathways, including cytochrome P450-mediated processes and platinum drug resistance, together with a hepatocellular carcinoma pathway. Collectively, these findings indicate that Midn deficiency preferentially perturbs coordinated hepatic metabolic and detoxification networks, particularly those involving membrane transporters, xenobiotic processing, and lipid metabolism.

### 3.3 Midn loss in female liver alters metabolic pathways and leads to sex-specific transcriptional responses

**Fig. 3:**
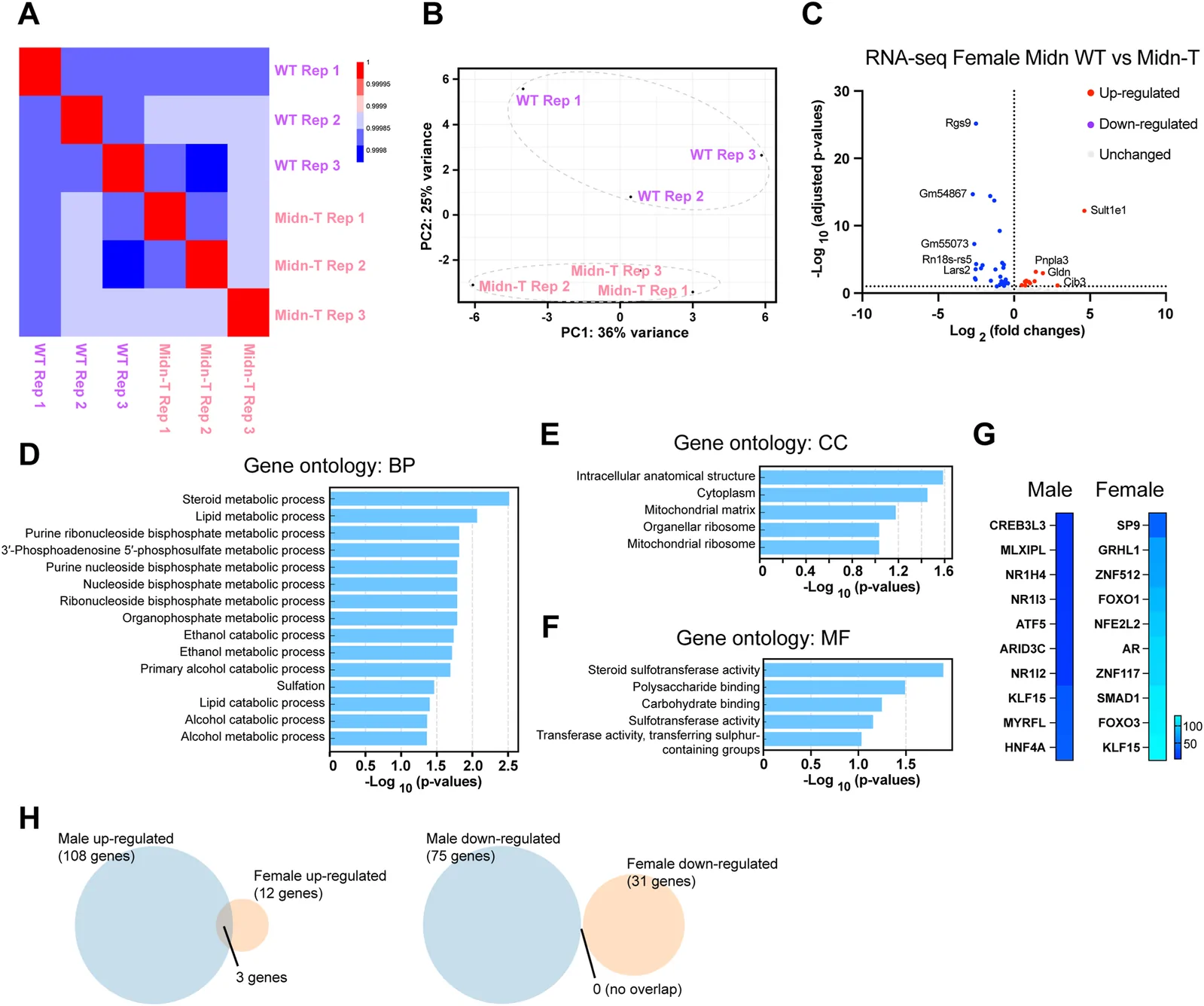
Transcriptomic effects of Midn-T in female liver **(A)** Sample correlation heatmaps showing overall consistency among biological replicates and clear WT–Midn-T separation. RNA-seq was performed using three biological replicates per genotype in female mice. **(B)** PCA plots showing separation of WT and Midn-T samples along PC1 and PC2 (36% and 25% variance, respectively). **(C)** Volcano plot illustrating differential expression between WT and Midn-T females. **(D-F)** GO enrichment analysis of differentially expressed genes in Midn-T female liver. Enriched GO BP, CC, and MF categories are shown. **(G)** Predicted upstream transcription factors identified by ChEA3 analysis for male and female Midn-T livers. **(H)** Venn diagrams comparing differentially expressed genes between male and female Midn-T livers. The left diagram shows the overlap of up-regulated genes, and the right diagram shows the overlap of down-regulated genes.

To determine whether Midn deficiency induces transcriptional changes in female liver similar to those observed in males, we performed RNA-seq on WT and Midn-T female mice. The correlation analysis demonstrated strong within-group clustering and clear separation between WT and Midn-T samples (Fig. 3A). The PCA analysis further highlighted genotype-dependent segregation, with PC1 explaining 36% of total variance and PC2 explaining 25% (Fig. 3B), confirming a distinct transcriptional signature associated with Midn deficiency.

Differential gene expression analysis revealed that Midn-T females displayed a modest but biologically meaningful set of transcriptional changes, with 12 genes up-regulated and 31 genes down-regulated at FDR < 0.1 (Fig. 3C). Several genes involved in metabolic and stress-response pathways were significantly up-regulated or down-regulated, consistent with the metabolic alterations observed in male Midn-T livers.

To identify biological pathways perturbed by Midn loss in Midn-T females, we performed Gene Ontology (GO) enrichment analysis on the differentially expressed genes. GO BP categories showed strong enrichment for small molecule metabolic processes, lipid metabolism, fat cell differentiation, and steroid metabolism (Fig. 3D). These pathways align with Midn's putative role in regulating hepatic metabolic gene networks. GO CC enrichment revealed that female DEGs encode proteins localized to the intracellular region, cytoplasm, mitochondrial matrix, and membrane-bound organelles (Fig. 3E). This pattern suggests broad effects on intracellular metabolic machinery, particularly mitochondrial functions associated with lipid and amino acid metabolism. GO MF categories highlighted alterations in transmembrane transporter activity, lipid transporter activity, and substrate-specific transporter activity (Fig. 3F), supporting the idea that Midn deficiency disrupts hepatic nutrient and metabolite transport.

Given Midn's function as a ubiquitin-independent proteasome adaptor, we next asked whether Midn loss may activate specific transcription factors whose stability is normally constrained by Midn-mediated degradation. ChEA3 transcription factor enrichment identified distinct sets of candidate upstream regulators in male and female livers (Fig. 3G). In male Midn depleted livers, enriched transcription factors included HNF4A, CREB3L3, MLXIPL (ChREBP), KLF15, and multiple nuclear receptors involved in metabolic and detoxification pathways (NR1H4/FXR, NR1I3/CAR, and NR1I2/PXR). These factors are central regulators of hepatocyte identity, nutrient sensing, bile acid metabolism, and xenobiotic response, indicating that Midn loss in male liver preferentially impacts core metabolic and transporter-associated transcriptional programs. In contrast, female Midn-T livers showed enrichment of transcription factors associated with stress-responsive signaling, hormone action, and dynamic transcriptional regulation, including FOXO1, FOXO3, NFE2L2 (NRF2), AR, and SMAD1, as well as factors linked to epithelial or differentiation-associated programs (GRHL1, SP9, and ZNF family members). These regulators are known to control oxidative stress responses, hormone-dependent transcription, and context-dependent cell-state transitions, suggesting that Midn loss in female liver engages transcriptional networks distinct from those observed in males. These findings suggest that the molecular consequences of Midn loss are largely sex specific and influence distinct regulatory networks in male and female liver.

To directly compare the transcriptional responses to Midn loss between sexes, we examined the overlap of differentially expressed genes identified in male and female livers. Only three up-regulated genes were shared between the male and female DEG sets, whereas no overlap was observed among down-regulated genes (Fig. 3H). Thus, although Midn deficiency perturbs metabolic pathways in both sexes, the specific downstream transcriptional responses are largely distinct between male and female livers.

### 3.4 Midn loss in female liver alters chromatin accessibility and selectively enriches metabolic transcription factor motifs

**Fig. 4:**
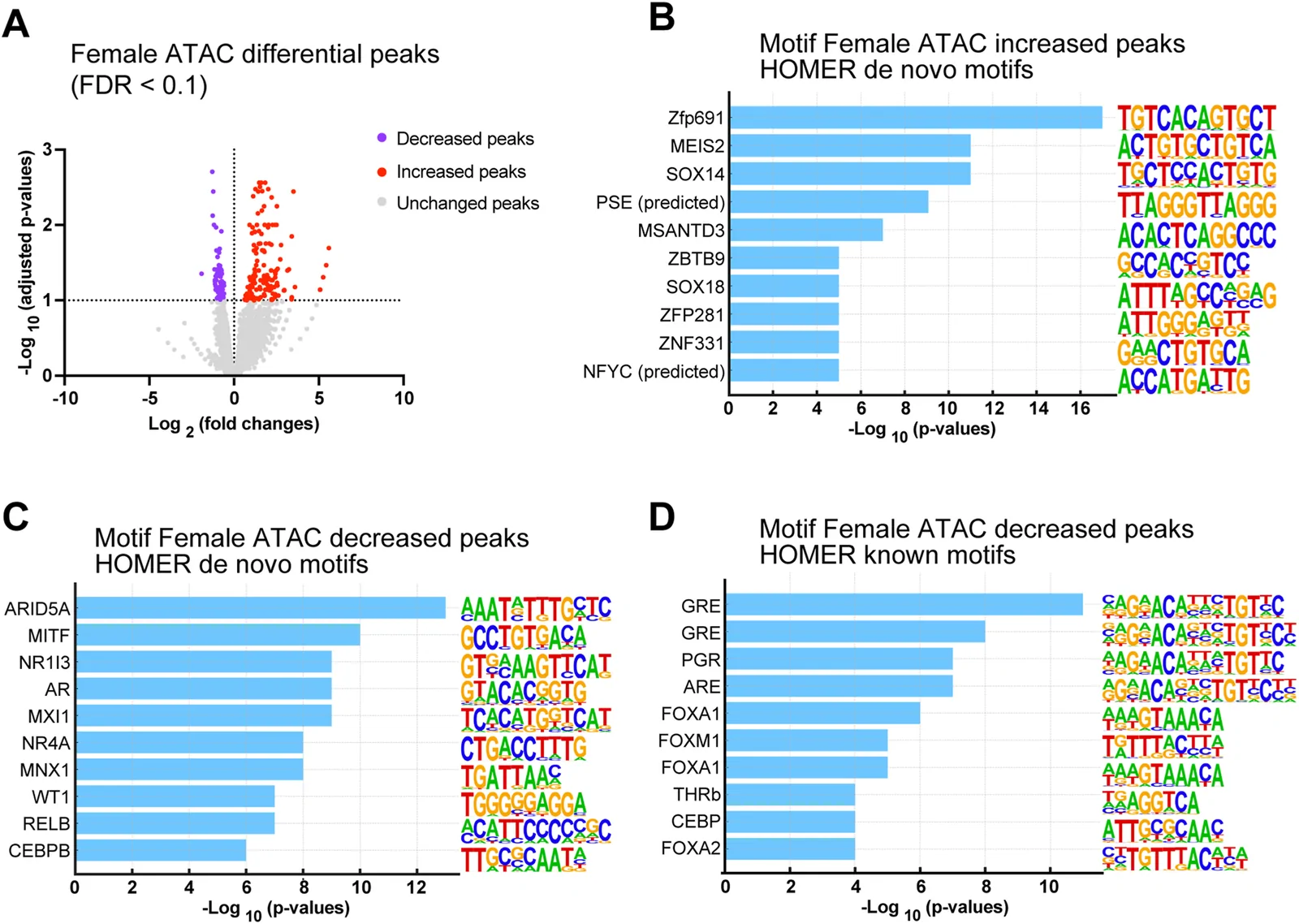
Chromatin accessibility changes and motif enrichment in female Midn-T liver **(A)** Differential ATAC-seq peak analysis in female livers (FDR < 0.1). ATAC-seq was performed using two biological replicates per genotype in female mice. Volcano plot showing numbers of increased and decreased peaks**. (B)** HOMER de novo motif enrichment for ATAC peaks increased in Midn-T females. **(C)** HOMER de novo motif enrichment for decreased ATAC peaks. **(D)** HOMER known motif enrichment for ATAC peaks decreased in female Midn-T, identifying motifs linked to nuclear receptor, FOXAand C/EBP family transcription factors.

To determine whether Midn affects chromatin structure in the liver, we performed ATAC-seq on WT and Midn-T female mice. Differential peak analysis (FDR < 0.1) showed that Midn depletion caused both gains and losses of chromatin accessibility, with a larger proportion of peaks exhibiting increased accessibility in Midn-T samples (Fig. 4A). These findings suggest that Midn may influence chromatin accessibility at a subset of genomic loci.

We next examined transcription factor (TF) motif enrichment at regions with altered accessibility. HOMER de novo motif analysis of ATAC-increased peaks revealed strong enrichment for motifs associated with Zfp691, MEIS2, Sox12, and several ZBTB/ZNF family factors (Fig. 4B). These TFs are frequently linked to developmental and metabolic gene regulation, suggesting that Midn loss may indirectly activate compensatory or stress-response transcriptional programs. In contrast, regions with decreased accessibility were enriched for motifs associated with nuclear receptor signaling pathways. De novo motifs included ARID5A, MITF, NR1I3, AR, and other TF motifs (Fig. 4C). Consistent with these findings, known motif enrichment identified GRE, PGR, AR, FOXA1/FOXA2, and C/EBP motifs among the most significantly enriched within ATAC-decreased peaks (Fig. 4D). These TFs play key roles in lipid handling, steroid hormone response, and hepatic metabolic regulation. Together, these results demonstrate that Midn regulates chromatin accessibility in female liver, particularly at loci bound by nuclear receptor and FOXA family transcription factors. The selective loss of accessibility at these regulatory motifs suggests that Midn may support transcriptional programs governing metabolic homeostasis by modulating chromatin openness at TF-bound enhancers.

### 3.5 Exploratory chromatin accessibility analysis identifies putative regulatory trends in male liver

Because ATAC-seq changes appeared less robust in male compared to female liver, we first assessed genome-wide accessibility differences between WT and Midn-T males. No differential peaks met an FDR < 0.1 threshold, indicating that chromatin accessibility changes in male liver are subtle or exhibit greater biological or technical variability relative to females. To capture potentially meaningful biological signals, we performed an exploratory analysis using a raw *P*-value cutoff of 0.05, which identified 102 peaks with increased accessibility and 77 peaks with decreased accessibility in Midn-T males. These exploratory datasets were subsequently used for unbiased motif enrichment and GSEA, with the understanding that results reflect trends rather than statistically stringent conclusions.

**Fig. 5:**
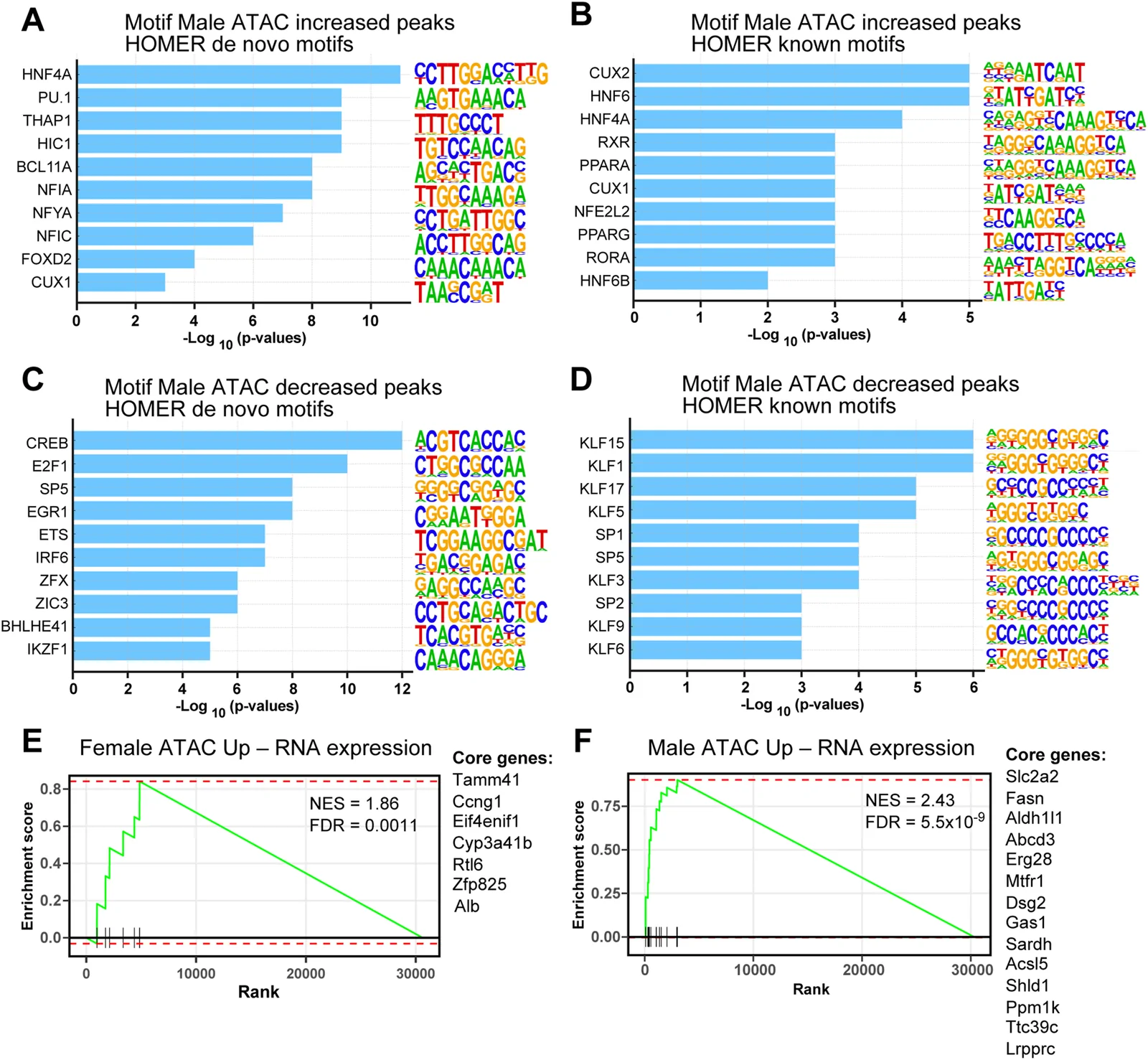
Chromatin accessibility changes and motif enrichment in male Midn-T liver. **(A-B)** HOMER de novo and known motif enrichment for ATAC peaks increased in male Midn-T liver, identifying HNF4A, RXR, PPAR, and related liver-enriched TF motifs. Male ATAC-seq data were generated from two biological replicates per genotype. **(C-D)** HOMER de novo and known motif enrichment for male ATAC decreased peaks, showing significant enrichment for CREB, E2F1, Klf15, and KLF1. **(E)** GSEA plot linking female ATAC-increased peaks to RNA expression changes. The gene set shows strong positive enrichment (NES = 1.86, FDR = 0.0011). **(F)** GSEA for male ATAC-increased peaks (NES = 2.43, FDR = 5.5 × 10−9).

Despite the limited number of confidently changing regions, motif analysis of ATAC-increased peaks revealed biologically coherent signatures. De novo motif analysis identified overrepresented motifs corresponding to HNF4A, PU.1, THAP1, BCL11A, NFIA, and NFY-family transcription factors (Fig. 5A). Known motif enrichment further highlighted Cux2, HNF6, HNF4A, RXR, and PPAR-family motifs (Fig. 5B), all well-established regulators of hepatocyte identity and metabolic function. Although exploratory, this enrichment pattern suggests that Midn loss increases accessibility at regulatory loci bound by key liver metabolic transcription factors. Conversely, ATAC-decreased regions were enriched for motifs associated with stress-responsive, proliferative, and metabolic transcription factors. De novo motif analysis identified motifs linked to CREB, E2F1/E2F2, Egr1, ETS-family, Zic3, BHLHE41, and IKZF1 (Fig. 5C). Known motif analysis highlighted strong enrichment of KLF family motifs (including Klf15 and KLF1) among accessibility-decreased regions (Fig. 5D).

These findings suggest that Midn may help maintain chromatin accessibility at a subset of KLF-bound regulatory elements, although confirmation will require additional datasets due to the exploratory nature of the male ATAC-seq results.

To evaluate whether accessibility changes corresponded to transcriptional output, we performed GSEA using genes located near ATAC-increased or ATAC-decreased peaks. Because neither male nor female datasets showed statistically significant enrichment at the full gene-set level, we instead examined the leading-edge subsets, the genes contributing most strongly to the nominal enrichment curves. In female liver, the leading-edge genes associated with ATAC-increased peaks included *Tamm41*, *Ccng1*, *Eif4enif1*, *Cyp3a41b*, *Rtl6*, *Zfp825*, and *Alb*, several of which are involved in mitochondrial or metabolic regulation (Fig. 5E). Similarly, in male liver, the leading-edge subset included metabolic genes such as *Slc2a2*, *Fasn*, *Aldh1l1*, *Abcd3*, *Erg28*, *Mtfr1*, *Dsg2*, *Gas1*, *Sardh*, *Acsl5*, *Shld1*, *Ppm1k*, *Ttc39c*, and *Lrpprc* (Fig. 5F). Although these leading-edge gene patterns do not reflect statistically significant enrichment at the pathway level, they highlight that genomic regions showing increases in accessibility may be associated with metabolic gene activation. Thus, although Midn loss produced only modest alterations in chromatin accessibility, the loci exhibiting changes remain linked to metabolic pathways central to hepatocyte function.

## 4 Discussion

In this study, we investigated the role of Midn in hepatic gene regulation by combining conditional genetic deletion with transcriptomic and chromatin accessibility profiling. Although Midn has been implicated in ubiquitin-independent proteasomal degradation and transcriptional regulation in other tissues, its physiological function in the liver has remained unclear. Our findings suggest that Midn contributes to hepatic transcriptional programs, particularly those involved in metabolic regulation, although its effects appear modest in young adult liver.

RNA-seq analysis showed that Midn loss induces reproducible but relatively small transcriptional changes in both male and female liver. Despite the limited number of differentially expressed genes, these changes segregated strongly by genotype, suggesting a coherent regulatory response to Midn deficiency. Many affected genes were associated with lipid metabolism, bile acid transport, and steroid or organic compound metabolism, processes central to hepatocyte function. Pathway enrichment analyses further highlighted Midn's contribution to hepatic metabolic networks and membrane-associated transporter systems.

Chromatin accessibility profiling revealed sex-specific differences in Midn-dependent regulation. In female liver, Midn deficiency produced clear sets of increased and decreased ATAC-seq peaks, which were subsequently examined by motif enrichment analysis. Peaks with reduced accessibility were strongly enriched for nuclear receptor motifs such as GRE/NR, PGR, AR, and FOXA family transcription factors, key regulators of bile acid metabolism, lipid handling, and hepatocyte identity. Conversely, ATAC-increased regions were enriched for Sox, MITF-related, and ZBTB/ZNF motifs, suggesting compensatory or stress-induced regulatory activation. These findings are consistent with a potential role for Midn in regulating chromatin accessibility at regions enriched for nuclear receptor motifs. In male liver, chromatin accessibility changes were more subtle due to either biological and/or technical factors. Exploratory analysis using a raw *P* < 0.05 threshold identified modest sets of increased and decreased peaks, which nonetheless showed biologically coherent motif enrichment patterns. Increased accessibility favored liver-enriched transcription factors such as HNF4A, HNF6, and PPAR family factors, while decreased accessibility favored CREB, E2F, and KLF family transcription factors.

A notable finding was the difference in the magnitude of the transcriptional and chromatin accessibility responses between males and females. Male Midn-T liver showed a slightly larger number of differentially expressed genes than differential chromatin accessibility changes, whereas female Midn-T liver showed fewer differentially expressed genes despite more changes in chromatin accessibility. One possible interpretation is that altered accessibility may reflect a permissive or primed regulatory state that does not necessarily produce corresponding changes in steady-state gene expression. Because Midn mediates the proteasomal degradation of selected transcription factors, the consequences of Midn loss may also depend on baseline Midn protein abundance and the levels of its target proteins. The limited overlap between transcription factors predicted by ChEA3 and motifs identified by HOMER likely reflects the distinct regulatory layers examined by these approaches. Nevertheless, several candidates were identified by both analyses, including HNF4A and KLF15 in male liver and AR in female liver. These shared candidates provide potential links between the transcriptional and chromatin accessibility datasets, although their protein abundance and genomic occupancy will require direct validation.

A key consideration that may explain the relatively mild chromatin and transcriptional consequences of Midn loss is the naturally low expression of Midn in young liver. Public transcriptomic resources, including GTEx, mouse aging studies, and the Tabula Muris Senis atlas, consistently show that Midn expression is low in young adult hepatocytes and increases with age. Because our analyses were performed in mice aged 11–14 weeks, when endogenous Midn expression is expected to be lower, the regulatory impact of its loss may be intrinsically attenuated. Under such conditions, Midn may not yet play a dominant role in maintaining hepatic transcriptional homeostasis, resulting in subtler phenotypic consequences upon deletion. This interpretation aligns with the small number of DEGs and the modest chromatin changes. Integration of RNA-seq and ATAC-seq further supported a connection between Midn and metabolic gene regulation. Although genome-wide GSEA did not yield significant enrichment, leading-edge inspection revealed metabolic genes, including *Slc2a2*, *Fasn*, *Aldh1l1*, *Erg28*, and *Alb*, associated with increases in chromatin accessibility. These patterns suggest that the regulatory regions most responsive to Midn loss map to metabolic pathways, even when global chromatin remodeling is mild.

Taken together, our findings suggest that Midn is a previously unrecognized modulator of hepatic metabolic gene expression whose functional impact may become more prominent with age. Midn loss is associated with transcriptional changes and modest accessibility alterations at regions enriched for metabolic transcription-factor motifs. The reported age-associated expression pattern of Midn raises the possibility that its regulatory contribution changes as hepatocytes age or encounter increasing metabolic and proteostatic demands. However, several limitations should be noted. The subtle chromatin changes in males and modest transcriptional responses across sexes warrant further investigation under conditions where Midn expression is naturally higher, such as in aged animals or metabolic challenge models. Direct evaluation of Midn protein levels in WT and Midn-T livers will also be important to determine whether Midn abundance differs between male and female mice or changes with age. Additionally, the specific nuclear proteins or transcription factors targeted by Midn for proteasomal degradation in hepatocytes remain unknown. Identifying these substrates will be essential for elucidating the mechanisms by which Midn integrates proteostasis with transcriptional regulation in metabolic tissues. Combining the inducible-reversible Midn-XTR gene-trap mouse model with tissue-specific depletion of expression using Cre recombinase alone or in combination with restoration of expression using Flpe recombinase will allow more refined analysis of the role of Midn in a variety of tissues and stages of development than constitutive Midn mutant models. In addition, the GFP reporter provides a practical marker for identifying and isolating cells in which the Midn-T allele has been activated. This feature may be particularly useful in tissues where endogenous Midn expression is low.

In summary, this study provides the first evidence that Midn is associated with hepatic chromatin and transcriptional regulation, particularly within metabolic pathways. These findings lay the groundwork for future mechanistic studies of Midn in liver physiology, aging, and metabolic disease.

## CRediT authorship contribution statement

**Lakshmi Sudeepthi Nissankararao:** Data curation, Formal analysis, Visualization, Writing – review & editing. **Gayeoun Kim:** Investigation, Validation, Writing – review & editing. **Soo-Mi Kweon:** Investigation, Validation, Writing – review & editing. **Vaisnavii Mohanraj:** Formal analysis, Visualization, Writing – review & editing. **Damien Parrello:** Formal analysis, Software, Writing – review & editing. **N. Sreekar:** Investigation, Validation, Writing – review & editing. **Sheng-Wei Chang:** Investigation, Validation, Writing – review & editing. **Walter Tsark:** Methodology, Resources, Writing – review & editing. **Keane Lai:** Conceptualization, Funding acquisition, Project administration, Supervision, Writing – review & editing. **Motoki Takaku:** Conceptualization, Data curation, Formal analysis, Funding acquisition, Methodology, Project administration, Supervision, Visualization, Writing – original draft, Writing – review & editing.

## Ethics declaration

This study was conducted in accordance with the ARRIVE (Animal Research: Reporting of In Vivo Experiments) guidelines. This study was approved by the City of Hope. (Approval No. 18153)

## Declaration of generative AI and AI-assisted technologies in the manuscript preparation process

During the preparation of this work, Takaku used ChatGPT (OpenAI) in order to improve the clarity and grammar of the manuscript. After using this tool, Takaku reviewed and edited the content as needed and takes full responsibility for the content of the published article.

## Financial support and sponsorship

This work was supported in part by National Institutes of Health (NIH) K08AA025112 to K.L. and by NIH R01GM148729 and P20GM104360 to M.T. The UND Genomics Core and D.P. were supported by NIH P30GM154634. S.-W.C. was supported by NIH 5T32CA221709. M.T. is a Research Scholar of the American Cancer Society (ACS Research Scholar Award, RSG-23-645952-01-DMC). Donations from Karol Endres to K.L. and the University of Southern California partially funded this work. M.T. has received research support from the University of North Dakota School of Medicine Health Sciences.

## Declaration of competing interests

The authors declare the following financial interests/personal relationships which may be considered as potential competing interests:Motoki Takaku reports financial support was provided by National Institutes of Health. Keane Lai reports financial support was provided by National Institutes of Health. Motoki Takaku reports financial support was provided by American Cancer Society. Keane Lai reports financial support was provided by Karol Endres. Damien Parrello reports financial support was provided by National Institutes of Health. Sheng-Wei Chang reports financial support was provided by National Institutes of Health. Keane Lai reports financial support was provided by University of Southern California. Motoki Takaku reports financial support was provided by University of North Dakota School of Medicine and Health Sciences. If there are other authors, they declare that they have no known competing financial interests or personal relationships that could have appeared to influence the work reported in this paper.

## Data Availability

All genomics data have been uploaded to GEO under GSE326870.
